# From Anomalies to Essential Scientific Revolution? Intrinsic Brain Activity in the Light of Kuhn's Philosophy of Science

**DOI:** 10.3389/fnsys.2017.00007

**Published:** 2017-02-28

**Authors:** Marek Havlík

**Affiliations:** Department of Applied Neurosciences and Brain Imaging, National Institute of Mental HealthKlecany, Czechia

**Keywords:** scientific revolution, reactive paradigm, intrinsic activity, resting state activity, default mode network, evoked brain activity, fMRI

## Abstract

The first step toward a modern understanding of fMRI resting brain activity was made by Bharat Biswal in 1995. This surprising, and at first rejected, discovery is now associated with many resting state networks, notably the famous default mode network (DMN). Resting state activity and DMN significantly reassessed our traditional beliefs and conventions about the functioning of the brain. For the majority of the twentieth century, neuroscientists assumed that the brain is mainly the “reactive engine” to the environment operating mostly through stimulation. This “reactive convention” was very influential and convenient for the goals of twentieth century neuroscience–non-invasive functional localization based on stimulation. Largely unchallenged, “reactive convention” determined the direction of scientific research for a long time and became the “reactive paradigm” of the twentieth century. Resting state activity brought knowledge that was quite different of the “reactive paradigm.” Current research of the DMN, probably the best known resting state network, leads to entirely new observations and conclusions, which were not achievable from the perspective of the “reactive paradigm.” This shift from reactive activity to resting state activity of the brain is accompanied by an important question: “Can resting state activity be considered a scientific revolution and the new paradigm of neuroscience, or is it only significant for one branch of neuroscience, such as fMRI?”

## Structure of scientific revolutions

It is impossible to deal with the question posed above without even a basic understanding of the terms *scientific revolution* and *paradigm*. American physicist and philosopher of science Thomas S. Kuhn brought to the philosophy of science one of the most fundamental concepts, which is often used not only by philosophers of science. This concept is a *paradigm* and it is tightly linked to Kuhn's hypothesis about the development of scientific knowledge. According to Kuhn's ideas, development of scientific knowledge is not continuous as one may presume, but is formed through major upheavals in scientific beliefs. Such major upheavals are called *scientific revolutions* that are accompanied by a change of the *paradigm*.

The paradigm determines the direction of research, it determines the problem and the methods to its solution and it also refers to the generally accepted beliefs, results and findings of the scientific community about the relevance and importance of the problem. The paradigm determines the so-called *normal science*, which is a specific period where scientists work under the weight of the current paradigm, and their research is characterized by the cumulating of relevant data. A cumulative process of research is intended to deepen and to shield the current paradigm rather than to seek out new problems or topics that could be investigated and researched. However, during this cumulative process special kinds of phenomena begin to appear–*anomalies*. These phenomena cannot be explained by the existing paradigm and for some time are usually ignored. After some time, however, repeatedly observable anomalies that resist scientific explanation begin to gain strength, and they lead to a period of *crisis*.

A period of crisis is caused by the inability of the paradigm (and the methods of normal science) to deal with the anomalies, which leads to the emergence of new hypotheses and theories that try to deal with anomalies and thus this period is characterized by the presence of many new theories that attempt to explain them. Only one of these new theories will become a generally acceptable solution and eventually a new *paradigm*. The process of replacement of one paradigm by another is called a *scientific revolution*.

This process of replacing one paradigm with a more adequate paradigm is repeated. Selection of a paradigm does not solely depend on rational values, but according to Kuhn it also depends on the social elements within the scientific community, which decide whether the new theory is adequate or not. Selecting a new paradigm, therefore, also includes negotiations in the scientific community, the strength of the scientific authority or the trendiness of the upcoming paradigm.

## Reactive paradigm

The so-called “*reactive paradigm”* began in the second half of the nineteenth century and was dominant during the twentieth century. “Reactive paradigm” could be roughly described as a belief that the functioning of the central nervous system is based mainly on reactive functions to the external environment (Llinás, 2002; Raichle and Snyder, 2007).

One of the first proponents of this model was James (1890/1950), who assumed that organization of the central nervous system is based reflexively. In this view, the brain is a tool that generates specific outputs to the inputs coming from the external environment. Sherrington (1906) also expected that the whole central nervous system is based on reflexive functioning and is, therefore, always busy with stimuli coming from the external environment. Some ideas resembling the reactive functioning, can also be found in works of *Santiago Ramón y Cajal*, the father of modern neuroscience. Cajal described several *laws* of nervous system, such as the *law of avalanche conduction*. This law says that even small but well-defined stimuli can cause the activation of large and distant populations of neurons to generate appropriate responses (Llinás, 2003; Delgado-García, 2015). Another is the *law of unity of spatial and tonal perception*. This law describes the need of central nervous system to generate integrated and united perception, which corresponds to the stimuli of the external world. Some ideas of reactive functioning could also be found in Cajal's interpretation of pyramidal cells. Pyramidal cells are capable of storing sensory information from the external world, which can be used for later responses (Delgado-García, 2015). Loeb (1912) can also be mentioned as one of the proponents of this reactive idea. During his scientific career, Loeb concluded that there is no free will and all animals including people are mechanisms, and their behavior is just a result of physical and chemical reactions to environmental stimuli.

Such ideas about the nature of brain functioning were very suitable for the former goal of neuroscience–*functional localization*, the revealing of the functions of the specific brain areas. We can mention works of *Paul Broca* and *Carl Wernicke* on expressive and receptive aphasia. Experiments performed by *Eduard Hitzig* and *Gustav Fritsch* with electrical stimulation of various areas of the dog's cerebral cortex, *Hermann Munk*'*s* localization of vision within the occipital cortex, *David Ferrier*'*s* localization of cortical areas responsible for motor functions and many others can also be mentioned. Further support of functional localization was also given by the formulation of the *neuron doctrine* (nervous system is made up of discrete individual cells) of Santiago Ramón y Cajal, whose ideas and experiments helped to abandon the *reticular theory* of Joseph von Gerlach and Camillo Golgi (Finger, 2001; Delgado-García, 2015).

In the second half of the twentieth century, invasive experiments on animals were largely replaced by non-invasive observation methods that finally allowed observation of the actual functioning of the human brain; however, the reactive idea and motivation of functional localization remained. Most of the knowledge and the conclusions about the functions of specific neural regions stem from non-invasive studies of the twentieth century. The methodology of these studies was based on correlations between neural activations and specific stimulation, cognitive or motor functions. The tradition of non-invasive studies and their wide success deepened the common belief that the brain is mainly a reactive instrument to the external environment.

Even though there were ideas that questioned the direct reactive functioning of the brain, other theories can be named that supported this belief. For example, the influential theory of American psychologist *James J. Gibson*. Gibson claimed that endogenously generated inferences are unnecessary postulates in normal perception and the external world, and its objects are perceived directly, not endogenously inferred. Influenced by radical empiricism, Gibson even claimed that the contents of cognition are the responses of the body to the world (Hochberg, 1994).

Similar tendencies of reactive functioning can also be found in the history of cognitive science. After the fall of behaviorism, which treated the mind as a “black box,” cognitive science was at its beginning deeply influenced by a computational approach to the mind and the brain. The computational approach or computational paradigm of cognitive science regarded the mind/brain as a computing system similar to the Turing machine, which operates through symbolic processing. The mind (and the brain) works on the basis of inputs (stimulation) and outputs (motor responses). In my view, the functioning of the mind and brain based on inputs and outputs with pure symbolic operations support the idea of “reactive paradigm.”

After two decades, around 1980, the computational approach to the mind (and the brain) was challenged by connectionism (although some argued that connectionism is absolutely compatible with computationalism). Connectionism moved away from computational ideas of the mind toward artificial neural networks, which consist of simple units and their links. The main strength of connectionism lies in the fact that it is close to the actual brain that consists of neurons and synapses. Networks can learn from their environment and generally represent natural mechanisms. It is not computation that is making the appropriate responses but various networks that cooperate with each other. Even though connectionism became more attractive than the computational approach the main idea of functioning remained the same–input and output. Building blocks of connectionism are processing units, *input units, output units*, and *hidden units*. Input units are understood as units responsible for receiving inputs from sources external to the network. Output units are responsible for the outcomes coming from the network and hidden units are those that communicate only with other units within the network. Connectionism introduced a much more natural way of thinking about the mind (and brain) than the computational approach; however, the main idea of the “reactive approach” largely remained untouched.

During this long scientific period of brain and mind, the neuroscientific community operated mostly within the context of *evoked brain activity* (stimulation–response/input–output) and *intrinsic brain states* were largely ignored.

## History of intrinsic brain activity

Intrinsic brain activity (also known as resting state activity) refers to the neural states that are produced spontaneously by the brain and not as responses to stimulation or immediate reactions to the environment. According to Raichle, intrinsic activity represents baseline neuronal activity or the so-called default mode of the brain associated with a high rate of energy consumption. Baseline in this context refers to the physiological baseline of brain activity, which is defined as the absence of activation–increase of blood flow (Gusnard and Raichle, 2001). Next to the baseline, no more than 5% of energy consumption is associated with reactive aspects of the brain functioning or so-called evoked brain activity (Raichle and Mintun, 2006; Raichle and Snyder, 2007; Raichle, 2009, 2015).

The whole scientific program of intrinsic activity started with Bharat Biswal and Gordon L. Shulman. Biswal's discovery of coherent resting activity of the sensorimotor cortex was based on his investigation of the various noise sources present in the brain and their changes between rest and task (Biswal, 2012). During the experiment, subjects went through several resting states and various forms of finger tapping (Biswal et al., 1995). Low-frequency signal was present in addition to the cardiac and respiratory signal, which was the first step to describing a significant correlation between the left and right sensorimotor cortices during rest (Biswal et al., 1995; Biswal, 2012). Conclusions of this paper had a significant impact, which was later visible in studies that demonstrated the resting functional connectivity of various sensory cortices (Hampson et al., 2002, 2004).

Independently of Biswal, Gordon L. Shulman et al. (1997) proposed two interesting ideas. *Firstly*, active tasks accompanied by blood flow increases apparently require consistent inhibition of the same areas (posterior cingulate/precuneus). *Secondly*, these decreases in activity during tasks with high probability lead to an absence of processes present during passive/rest conditions, such as monitoring of the external environment (later known as the *Sentinel* hypothesis), unconstrained thought processes (now known as various *self-referential* thoughts), monitoring body states, monitoring emotional states, and also creating the Self. According to Schulman's paper, one of the areas that showed consistent decreases during active tasks was the posterior cingulate/precuneus (Shulman et al., 1997). This area was nicknamed the medial mystery parietal area (Raichle and Snyder, 2007) and is now considered the central region of default mode network (DMN) (Fransson and Marrelec, 2008; Utevsky and Smith, 2014), probably the best known resting state network consisting of the posterior cingulate/precuneus and medial prefrontal cortex, which represent its core regions.

Since this time, many resting state networks have been identified along with their structural connectivity, such as parietal-frontal networks, the primary motor network, the visual network, extra-striate visual networks and others (see van den Heuvel et al., 2009). A great deal of attention is now focused on the recently discovered *salience network* (SN), which consists of the anterior insula and the anterior cingulate cortex. Main functions of the SN lie in “detecting salience stimuli” and “dynamical switching” between the other two large scale networks–the DMN and the *central executive network* (CEN), which is associated with evoked brain activity (Menon and Uddin, 2010). And also the recently described *parietal memory network* (PMN) consisting of the precuneus and the medial cingulate cortex. The PMN is supposed to be active when familiar stimuli are presented and deactivated when novel stimuli are to be remembered (Gilmore et al., 2015). But no other resting state network has made such significant impact on the neuroscientific community and helped establish resting brain activity as a prosperous neuroscientific theme as the DMN.

## Default mode network

The DMN has been an interesting and topical theme in neuroscience since the beginning of the twenty first century. Based on the PubMed search in 2015, more than 600 studies have been published that deal with the behavior of the DMN. This network is currently characterized as a large-scale resting brain network consisting of specific brain regions: *medial prefrontal cortex* (mPFC), *ventral medial prefrontal cortex* (vMPFC), *medial and lateral temporal cortex* (m/lTC), *posterior inferior parietal lobule* (pIPL), and *precuneus/posterior cingulate cortex* (PCC). The very nature of the DMN is that it is highly active and highly functionally connected during passive states that can be achieved by behavioral rest. The first idea of this resting state network was established based on regular observations of identical decreases in the activity of the mPFC and PCC when subjects went from a passive resting state into states of reactive or evoked cognition (Raichle et al., 2001). Reactive states are described as states of evoked activity that are induced by stimulation or are present during states of active attention, which is necessary for immediately responding to the demands of the environment. These states of evoked activity are bound to another large scale brain network, the so-called (CEN), which, by the nature of its activity, is in anti-correlation with the intrinsic activity of the DMN, meaning if one is active then the other is partially disengaged and vice versa (Gusnard and Raichle, 2001; Gusnard et al., 2001; Raichle et al., 2001; Raichle and Snyder, 2007). Interestingly, novel computation approaches to resting state fMRI (*still relatively new method of functional brain imaging used for the observation of spontaneous regional interactions, which occur when a subject is not performing an explicit task*) analysis were introduced at the same time. Methods, such as seed-driven functional connectivity or independent component analysis (ICA) allow us to identify and quantify resting brain networks (including DMN) in any fMRI time series even without the condition of task-dependent deactivation. Interesting observations were reported about the connectivity of the regions of the DMN. Connectivity of DMN is in fact formed successively through the development of the individual and within its ontogenesis (Fair et al., 2008). This fact was also demonstrated by an fMRI study focusing on infants. Fransson et al. (2007) found several resting brain networks; however, they failed to detect a direct equivalent of the DMN, hypothesizing that the absence of the DMN could be related to the immaturity of the infant brain (Fransson et al., 2007).

The *default mode of brain function* (Raichle et al., 2001) and the so-called *baseline* of neural activity (Gusnard and Raichle, 2001) were described in 2001. A study was published shortly afterwards focusing on the functional connectivity of *default mode*, which claimed that this intrinsic activity is in fact a highly connective functional brain network–the DMN (Greicius et al., 2003). This was quite surprising because until then only the motor and sensory networks had been discovered (by researches of the reactive paradigm) and the existence of resting state networks was almost entirely out of the question (Raichle and Snyder, 2007). In addition, the idea that deactivations of a passive state, used in most cases only for the contrast to the reactive experiments, would probably hide the most important network of the human brain was very unlikely. But soon after the studies showed that the DMN exists, it became a “matter of fashion” that could not be ignored by any neuroscientific laboratory. The fact that no special equipment is needed to measure resting states of the brain also played an important role. Subjects are only asked to stay in a state of behavioral rest. Furthermore, it was only a matter of time until intrinsic activity and the DMN were incorporated into ongoing research programs with various different objectives and themes. In this paper, we will describe these research programs with the assistance of Kuhn's philosophy, in order to answer the question whether the DMN is a new paradigm of neuroscience.

## The resting state and the DMN in the light of Kuhn's philosophy of science

Resting brain activity and the DMN became very interesting topics in twenty first century neuroscience. Marcus Raichle admitted that the DMN generated a much greater interest than he ever expected (Raichle and Snyder, 2007). In the following text, we will describe how the DMN and its subsequent research revolutionized our understanding of the brain and we will also analyze whether resting state activity corresponds to the model of Thomas S. Kuhn and whether it can be considered a new paradigm of neuroscience.

There are several conditions that a new paradigm has to fulfill. One of them is the *promise of success* of a new paradigm:

“*The success of a paradigm*–whether Aristotle's analysis of motion, Ptolemy's computations of planetary position, Lavoisier's application of the balance, or Maxwell's mathematization of the electromagnetic field–is at the start largely a *promise of success* discoverable in selected and still incomplete examples. Normal science consists in the actualization of that promise […] (Kuhn, 1962, pp. 23–24).”

The promise of success is essential for a new paradigm and the new paradigm has to immediately begin to fulfill such promise. In this case, the promise is related to the progress of our understanding of brain functioning and progress in themes that are yet to be explained. The condition of the success of a new paradigm has begun to be fulfilled immediately after the claim that intrinsic activity represents the baseline of neural activity (Gusnard and Raichle, 2001; Raichle et al., 2001). Describing the baseline of neural activity essential for any other brain activity is in itself a great success; however, the greatest promise of success of the DMN and intrinsic activity is through the acquisition of more knowledge about mental and neurodegenerative disorders. This was maybe the most vital reason why the DMN stole the fame from Biswal's original paper. For a long time, the neuroscientific community looked at the brain differently than it does now. Neuroscientists thus unintentionally neglected the important manifestations of mental disorders in the resting state activity of the brain. Currently, it is common knowledge that various disorders alter the functioning of the DMN. All of the studies that connect the DMN with psychiatric and neurodegenerative disorders describe atypical functioning of the DMN (Buckner, 2012), which manifests itself as an increased or decreased activity or functional integrity of the brain regions that form this resting state network. Important discoveries were made for Alzheimer's disease, schizophrenia and depression (see e.g., Greicius et al., 2007; Broyd et al., 2009).

Relative to controls, patients diagnosed with *Alzheimer's disease* have reduced DMN activity particularly in the PCC and medial temporal lobe (Greicius et al., 2004). Another resting state fMRI study presented interesting behavior of the right hippocampus in patients with Alzheimer's, namely its reduced functional connectivity of the nodes of the DMN (Wang et al., 2006). Resting state fMRI and observation of intrinsic activity can also be quite successful (another *promise of success*) for the early recognition of the onset of a neural disease. Early determination of neural biomarkers for various disorders is one of the most important things for further treatment. Individuals with mild cognitive impairment can be differentiated from healthy individuals by the degree of deactivation of the DMN regions MFG, precuneus/PCC and anterior cingulate gyrus (Rombouts et al., 2005). A similar differentiation between mildly cognitively impaired individuals and patients diagnosed with Alzheimer's disease can be made based on differences in the deactivation of the *anterior cingulate cortex* (Rombouts et al., 2005). Several authors also try to put the DMN into context with genetic predispositions to Alzheimer's disease. The hypothesis is that from a genetic perspective people with the risk of Alzheimer's diseases have a much lower ability to deactivate the DMN (Persson et al., 2008).

According to resting state study of patients diagnosed with paranoid *schizophrenia*, both large-scale networks, the DMN and CEN, exhibit increased connectivity. According to the study, such increased connectivity could hypothetically correlate with increased sensitivity to the external environment and self-referential thoughts (Zhou et al., 2007). Furthermore, an explanation of hallucinatory experiences during schizophrenia could be linked to the increased connectivity between the DMN and other resting state brain networks (Jafri et al., 2008). Wang et al. (2015) claim that the DMN plays a pivotal role in schizophrenia. By dividing the DMN into three subsystems, i.e., anterior, posterior and lateral DMNs, they were able to observe atypical connectivity between various networks and subsystems of DMNs. For example, intra-default functional connectivity between the anterior and lateral DMNs was significantly increased in schizophrenic patients, and lateral DMN exhibited decreased functional connectivity with unimodal cortical networks and increased functional connectivity with heteromodal cortical networks. Lateral DMN also showed increased functional connectivity with the right control network, which significantly correlated with PANSS (positive and negative syndrome scale) negative and, furthermore, lateral DMN exhibited significant functional connectivity decreases with unimodal systems, including the somatomotor, motor, lateral visual, and auditory networks, in schizophrenic patients. According to the results of the study, based on the DMN connectivity patterns, schizophrenic patients can be differentiated from normal subjects with 76.9% accuracy (Wang et al., 2015).

The resting state and the DMN also exhibit atypical behavior in patients with *major depression*. According to a resting state study, the higher activity of the subgenual cingulum negatively contributes to the natural activity of the DMN. The increases in activity are shown to be proportional to the length of the depressive episode. In addition, higher connectivity of the thalamus and limbic system was observed in individuals diagnosed with major depression. Authors assume that these factors have a negative influence on the brain areas responsible for the various cognitive processes (Greicius et al., 2007), and that hyperactivity of limbic regions could be caused by reduced effective connectivity from the anterior subsystem to the ventral subsystem of the DMN (Sambataro et al., 2013).

But these three most widespread disorders are not the only themes to connect psychopathology with the atypical functioning of the DMN and intrinsic activity. Various DMN dysfunctions were found in patients with *Parkinson's disease*. A comparison between patients with Parkinson's disease and healthy controls showed a decrease in the functional connectivity of the right medial temporal lobe and the bilateral inferior parietal cortex within the DMN (Tessitore et al., 2012). Individuals diagnosed with *ADHD* are unable to suppress DMN activity during most of the test trials (Metin et al., 2015). Several studies demonstrated that individuals with *autism* have lower functional connectivity between frontal parts of the DMN, PCC and MTL (core regions of the DMN) compared to healthy individuals (Monk et al., 2009; Weng et al., 2010; Menon, 2011; Jung et al., 2014). Large decreases of functional connectivity across many large-scale brain networks, including the precuneus (the central region of the DMN), were reported in patients with *fragile X syndrome* (Hall et al., 2013).

Despite the fact intrinsic activity can be related to many disorders, its role in medical diagnosis and medical practice is currently a modest one. It is not only the problem of intrinsic activity but the entire fMRI, which is still seldom used in medical practice. The use of fMRI is growing in the context of presurgical planning, but in the routine medical practice, such as diagnosis of psychiatric disorders, the use of fMRI as a diagnostic tool is still largely missing (Rosen and Savoy, 2012).

Another promise of success of intrinsic activity and the DMN can be seen in studies of *consciousness*, which still represents the most enigmatic topic in neuroscience, neurophilosophy and the philosophy of the mind. Recent findings show that the activity of regions of the DMN is in direct correlation with levels of consciousness. Connectivity of the DMN reflects the level of consciousness, ranging from healthy controls to minimally conscious, vegetative and comatose patients (Vanhaudenhuyse et al., 2010). Success in revealing information about the DMN and its relation to the consciousness leads some to the idea that the intrinsic activity of the brain could be used to finally resolve the enigmatic problem of the conscious experience.

Consciousness is not the only theme of philosophically oriented questions of the mind in which the DMN promises success. Role of the DMN is also considered in the *ongoing mental phenomena* that are present during intrinsic activity. However, research of mental phenomena has been significantly delayed by the definition of the DMN. The DMN was characterized as a resting brain network, which decreases its activity when the subject enters into a state of active cognition. Such a definition of intrinsic activity has led to the general belief that if the subject began to produce any kind of active cognition, it would interrupt intrinsic neural activity. “It lulled us into focusing on the default network's attenuation during most active tasks, and it took several years before ideas on the default network's role in active cognition were brought to the forefront. And that's when things got really interesting” (Buckner, 2012, p. 1142). After this, various researches determined that intrinsic activity is in relation to *self-referential thinking* (Gusnard and Raichle, 2001), *imagining the future* (Buckner et al., 2008), *spontaneous cognition* (Andrews-Hanna et al., 2010). *mind wandering* (Mason et al., 2007), *remembering the past* and *imagining the future* (Addis et al., 2007), *mental time travel* (Østby et al., 2012), *social cognition* (Mars et al., 2012), *theory of mind* (Buckner et al., 2008; Nekovarova et al., 2014), *internal train of thought* produced by cooperation between the DMN and frontal-parietal network (Smallwood et al., 2012) etc., which have the overall character of spontaneous imagination or daydreaming. Current interest lies in the questioners of spontaneous cognition that try to capture the form and content of these mental states (Delamillieure et al., 2010; Diaz et al., 2013, 2014; Gorgolewski et al., 2014).

The questions of *neural etiology* began to arise after the claim that activity of the DMN represents a functional baseline of neural activity. The first question was: which region is the central region of the DMN? From the onset, the precuneus was the main candidate because it takes about 35% more glucose than other neural regions in the brain, which puts it in first place on the scale of metabolic activity of the DMN (Gusnard and Raichle, 2001). Nowadays, most contemporary neuroscientists consider the precuneus/PCC to be the central region of the DMN (Fransson and Marrelec, 2008; Utevsky and Smith, 2014). Another question of neural etiology arose a few years later. In this case, the focus was on the relation between two anti-correlated brain networks, the DMN and the CEN. Research has shown that the function of the SN is to find the salient stimuli and subsequently deactivate the DMN and activate the CEN, which was described as “dynamical switching” between these two large scale networks (Menon and Uddin, 2010). Further success of the DMN can be seen in the subsequent research that put together the SN, DMN and CEN and led to the formulation of the so-called *Triple network theory*. The triple network theory also promises success in answering questions about psychical disorders because dysfunction of its mechanisms is considered to be the underlying condition of several neurological and psychiatric disorders (Menon, 2011; Nekovarova et al., 2014).

The DMN also promises some success in the topic of *localization* of specific ongoing mental states. An interesting study was performed by Demis Hassabis. The goal of this particular experiment was to show where modules for people and their personalities are stored. Experiment was based on the predicting behavior of specific people in specific locations during the phases of resting states. The results showed that personalities of other people can be localized in the medial temporal lobes, the posterior inferior parietal lobule, the PCC, and mPFC–central nodes of the DMN (Hassabis et al., 2013).

The various successes lead us to ask whether the DMN is solely a human neural system. Recent findings documented that the DMN can also be found in monkeys and rodents (Mantini et al., 2011; Lu et al., 2012), which could have important implications for evolutionary biology in the future.

*Promise of success* is not the only thing that defines a new paradigm. Scientific revolution is accompanied by a change in the “neuroscientific worldview.” We can never look at the brain in the same way as before–only from the perspective of reactive brain functioning.

In his work, Kuhn says:

“Each (scientific revolution) produced a consequent shift in the problems available for scientific scrutiny and in the standards by which the profession determined what should count as an admissible problem or as a legitimate problem-solution. *And each (scientific revolution) transformed the scientific imagination in ways that we shall ultimately need to describe as a transformation of the world* […]” (Kuhn, 1962, p. 6).

and

“(W)hen paradigms change, the world itself changes with them. Led by a new paradigm, scientist adopt new instruments and look in new places. Even more important, *during revolutions scientists see new and different things when looking with familiar instruments in places they have looked before*. […] (*P)aradigm changes do cause scientists to see the world of their research-engagement differently*. […] What were ducks in the scientist's world before the revolution are rabbits afterwards” (Kuhn, 1962, p.111).

In the previous section, we described a reactive paradigm that was based on the widespread conviction that the brain is a mainly reactive engine to the environment. Research of reactive brain functions brought important conclusions about functional localization and its success unintentionally caused the neglect of the resting states. The discovery of intrinsic brain activity significantly updated our research of brain functioning. According to several authors, reactive experiments revealed only a small portion of the actual brain functioning (Gusnard and Raichle, 2001; Raichle et al., 2001; Raichle and Snyder, 2007). When neuroscientists now look at the brain they do not primarily see reactive functions of the brain. Their world came through the crucial transformation that is now clearly visible in the acceptance of the fact that the brain has significant intrinsic activity. Revolutionary changes can be seen in the current neuroscientific research programs, and also can be observed explicitly in the critical language of current neuroscientists, who openly speak about the long-term omission of resting brain states. It is possible to cite several authors that share this conviction. Samantha Broyd on one occasion said: “For researchers to focus on active task-oriented conditions to the exclusion of rest may be a significant oversight” (Broyd et al., 2009, p. 280). Marcus Raichle also said that: “Unfortunately, the success of studying evoked activity has caused us to lose sight of the possibility that our experiments reveal only a small fraction of the actual functional activity performed by our brain” (Raichle, 2010, p. 180).

An important part of Kuhn's philosophy is played by *anomalies*:

“New and *unsuspected phenomena* are, however, repeatedly uncovered by scientific research, […]” (Kuhn, 1962, p. 52).

The very idea of the existence of intrinsic brain activity was based on the observation of a specific recurring *anomaly*. It is possible to attribute the status of the anomaly to the dominant source of noise (low frequency fluctuations), which Biswal observed as an addition to the cardiac and respiratory signal. The finding such an anomaly was not received with enthusiasm. Biswal (2012) in his personal review says that “one audience member (who shall remain nameless —but who is now heavily involved in resting-state connectivity research!) suggested that I, along with my research, should be buried since this would destroy fMRI.” This corresponds with Kuhn's idea that *novelties* are not welcome during the phase of *normal science*.

In the case of Shulman et al. (1997) and Raichle, anomalies can be connected to the recurring decrease of activity in the posterior cingulate/precuneus, which was called the *medial mystery parietal area* (Raichle and Snyder, 2007). Decreases of activity in the medial mystery parietal area were related to what is now known as the anti-correlate relation between the CEN and DMN, observable in moments when the subject goes from a passive resting state to states of reactive or experimentally stimulated cognition. However, the phenomenon of repeated reduction in neural activity was not an isolated observation. Most laboratories commonly observed such a deactivation, but these data have been systematically overlooked as is natural for the phase of normal science. This anomaly, which became the first step toward the description of the DMN, had no vital interpretation in the “reactive” paradigm. Since the previous tradition focused primarily on reactive aspects of neural activity, these frequent anomalies were unintentionally but largely overlooked. Randy L. Buckner mentioned in his review how the absence of clarification of this anomaly did not allow researchers to see the activity of the DMN: “Anyone conducting a human neuroimaging study that uses a passive baseline as a control will observe the default network if they look […]. We documented its presence across four different contrasts but, having little grasp of its importance only noted, ‘Although the fixation task was intended as a low-level control task, it nevertheless may require distinct processing resources”’ (Buckner et al., 1995; Buckner, 2012, p. 1138).

Kuhn also says about the new paradigm:

“[…] new paradigm must promise to preserve a relatively large part of the concrete problem-solving ability that has accrued to science through its predecessors. Novelty for its own sake is not a desideratum in the sciences as it is in so many other creative fields. As a result, though *new paradigms* seldom or *never possess all the capabilities of their predecessors, they usually preserve a great deal of the most concrete parts of past achievement and they always permit additional concrete problem-solutions besides*” (Kuhn, 1962, p. 169).

Nevertheless, the resting state does not exclude or naively falsify the results and observations of previous studies that fall under the “reactive” paradigm. The arrival of intrinsic activity and the DMN and their incorporation into specific research programs in no way precluded observations of reactive activations of brain regions. Furthermore, intrinsic activity is not tied to a specific method of measurement. It is observed in the same manner as brain functions that require active attention and evoked activity, i.e., using non-invasive methods, such as functional magnetic resonance imaging or positron emission tomography. Intrinsic activity retains the results of the previous paradigm, which can be easily replicated using cognitive tasks requiring active attention. Therefore, it could be considered that reactive functions of the brain are explainable from specific resting conditions that constitute an integrated neural baseline. Kuhn says that: “Though *an out-of-date theory can always be viewed as a special case of its up-to-date successor*, it must be transformed for the purpose” (102–103). The question as to whether resting state/intrinsic brain activity is a new paradigm of neuroscience could be answered simply if there was a universal consensus on looking at evoked brain activity as a special case of intrinsic brain activity. Raichle showed that the functioning of the *baseline* is related to high energy consumption and according to him evoked brain states exceed this high-energy consumption by no more than 5% (Raichle and Mintun, 2006; Raichle, 2009, 2015). Raichel's finding could be used to support the idea that evoked brain states are actually special cases of baseline activity.

“(T)here can be *small revolutions* as well as *large ones*, […] some revolutions affect only the members of a professional subspecialty, and that for such groups even the discovery of a new and unexpected phenomenon may be revolutionary. […] For the rest of the profession and for those who practice other physical sciences, that change need not be revolutionary at all” (Kuhn, 1962, pp. 49–50).

There is no question that the resting state had a revolutionary impact on neuroscience. Its research brought new knowledge of various neuropsychiatric disorders, functional connectivity processes and brought attention to the spontaneous mental phenomena. Various resting networks were discovered with their structural connections, and a correlation between the DMN and various levels of consciousness was also shown. According to some, resting state functional connectivity is highly reliable tool for gathering valuable pre-surgical information. It can be used during a patient's sleep or sedation and thus does not require the patient's cooperation, which is crucial for evoked fMRI (Shimony et al., 2009). Others even consider the resting state to be a more proficient tool than evoked brain activity for revealing more functional connections (Xiong et al., 1998, 1999; Biswal, 2012).

Under the pressure of these ideas, various new discoveries described in this review and with a seductive metaphor that evoked activity represents only the tip of the iceberg of the total activity of the brain, it is possible to state that intrinsic activity was a large scientific revolution and a new paradigm of neuroscience. However, this idea is not tenable at all.

## Against the idea of scientific revolution

Even though some aspects of resting activity correspond well with several of Kuhn's ideas, there are some elements of Kuhn's philosophy that intrinsic activity does not satisfy.

During the period of the *reactive paradigm*, neuroscientists concentrated on their own research of reactive aspects of brain areas. During this period, the *anomalies* (noise/low frequency signal, deactivations of the medial “mysterious” parietal area) were overlooked in most cases, which is typical for a phase of *normal science* that does not identify *novelties*. However, recurring *anomalies* did not induce a phase of *crisis* in *normal science* and did not present a major problem that would threaten the *reactive paradigm*. Observable anomalies, therefore, did not spark a scientific revolution. Resting state activity and the DMN came to neuroscience as an explanation of these anomalies and not as the winners of a *crisis* in neuroscience.

Furthermore, another aspect of the scientific revolution did not appear. According to Kuhn, the inability of the former paradigm to deal with anomalies leads to the emergence of new hypotheses and theories. By explaining anomalies, these new theories strive to become the new paradigm. Nothing like this happened in the short history of intrinsic activity. There was no emergence of another hypothesis and the DMN was not one of the many theories that would try to explain observable anomalies. Thus, again, resting state activity and the DMN cannot be considered as the winners in the competition of the various explanatory hypotheses because such hypotheses were not even formulated.

On the basis of this analysis, one should adopt a skeptical attitude toward the idea that intrinsic activity is a fully fledged scientific revolution and a new paradigm of neuroscience. On the other hand, taking into account the success, progress and deepening of knowledge about the brain due to intrinsic activity (and the DMN), it is seductive to conclude that resting brain activity presents a new paradigm of neuroscience even though it does not exactly match the phases described by Kuhn. Now, an interesting question should be asked: is it possible to consider intrinsic activity as a significant scientific revolution even though it happened without the phase of crisis in the normal science of a reactive paradigm? I propose the following: intrinsic activity could be understood as a new paradigm of neuroscience if and only if two conditions are satisfied. *Firstly*, there has to be a universal consensus that understands any evoked brain state as a *special case* of intrinsic activity and that any such state is explainable from states of intrinsic activity. *Secondly*, intrinsic activity had to absolutely break from the history of neuroscience and establish a completely new and unexpected perspective on how we look at the brain.

### Condition 1

The previous section described how baseline activity has a high rate of energy consumption and evoked brain states exceed this consumption by no more than 5% (Raichle and Mintun, 2006; Raichle, 2009, 2015). For some thinkers, it could be seductive to claim that any activation is a special case of resting state activity. However, it is very unlikely that the neuroscientific community would reach a consensus on understanding any sensory, motor, emotional or higher cognitive function or even subjective conscious experience as only a special case of intrinsic activity. It would just stand against natural intuition. Most probably there are proponents of the idea that all the resting state and the DMN represent is that everyone doing fMRI has the same noise, same background fluctuations in their signal, and everyone analyzes their data with this in mind. Neuroscientists have just come to understand their signals better. Although it is certainly progress, it is nothing more than clarification of the signal and therefore it would be highly misleading to think about the resting state as a new paradigm of neuroscience. For example, Morcom and Fletcher (2007) severely criticized resting brain activity and the concept of the DMN. The authors stated that the study of rest is not the right choice for studying the functions of neural regions. Although resting state activity is interesting, it does not have any special significance. Rather than focusing on rest, neuroscience should focus on creating more well-designed cognitive tasks for studying cognition.

It is highly unlikely that evoked brain states (and evoked activity) would only be understood as a special case of resting activity. Furthermore, intrinsic activity was never meant to falsify and replace reactive aspects of the brain in the first place. It is much more pragmatic and also natural to claim that a reduction of evoked brain states to intrinsic brain activity is out of the question and evoked brain states stand next to rest and intrinsic brain states. This leads to the conclusion that the first condition cannot be satisfied.

### Condition 2

In the twentieth century the common belief, now referred by many as the central dogma of neuroscience, was that the adult brain cannot produce any new neurons. In the second half of the twentieth century several scientists tried to falsify this deeply rooted neuroscientific belief. First one was Altman (1962) who showed the evidence of the existence of newly created neurons in the adult rodent brain. Over the years new neurons were found in brains of several adult animals, such as rats (Altman, 1962; Kaplan and Hinds, 1977), cats (Altman, 1963), Guinea-pigs (Altman and Das, 1967) and Canary birds (Goldman and Nottebohm, 1983). However, the dogma was still not fully falsified due to the idea that the proliferation of new neurons is impossible in adult brains of higher mammals, such as primates (and humans) (Rakic, 1985). It took the pioneers of neurogenesis (e.g., Elizabeth Gould, Fred Gage, Peter S. Eriksson) another decade to overcome this idea. In 1997, relation between enriched environment and new neurons in dentate gyrus of adult mice was shown (Kempermann et al., 1997). Later the effects of exercise (running) on the proliferation of cells in dentate gyrus of adult mice were described (van Praag et al., 1999). In 1998, Peter S. Eriksson and Fred Gage were the first who documented the existence of new neurons in the hippocampi of several adult human brains (Eriksson et al., 1998) and 1 year later, Elizabeth Gould was the first one who proved the existence of new hippocampal neurons in adult *Old World primates* (Gould et al., 1999a). In the same year, she reported the existence of new neurons in neocortex of adult *Macaca fascicularis* (Gould et al., 1999c) and also showed that the number of new cells increases in dentate gyrus of adult rats in response to associative learning (Gould et al., 1999b).

Over the years, this dogma of *no new neurons in adult brain* has been overcome and replaced by a new paradigm that can be described as a paradigm of “adult neurogenesis.” A paradigm of adult neurogenesis is the absolute opposite of its predecessor–the adult brain is capable of producing new neurons and these are born constantly. Revolutions in neuroscience are associated with a radical reassessment of unquestioned beliefs about the brain and its functioning. Any such revolution subsequently leads to progress and success because it significantly deepens our knowledge and can lead to the solution of problems that resisted scientific endeavor for far too long due to the persistence of the previous paradigm. The new paradigm has to radically break from the history of its predecessor.

Does intrinsic activity radically break from the history of neuroscience and was the reactive paradigm so persistent that it would not allow any ideas of intrinsic or endogenous brain activity? No. There were several ideas that opposed the view of the simple reactive functioning of the brain. For example, *Hermann von Helmholtz* claimed that visual perception is not direct. He assumed that visual perception is the result of unconscious inferences from sensory data and knowledge acquired in the past. Perception is therefore not “directly” related to the external environment, but it is created endogenously (Gregory, 1997). Sherrington's own student *Thomas G. Brown* broke with ideas of his mentor when he concluded that the nervous system is not driven reflexively. He based his belief on observations of animals with severed afferent pathways. These animals were still able to generate locomotion even though their afferent pathways were severed. Such observations led him to the conclusion that the external environment is not the initiator of these functions and thus the movement is produced endogenously and not as a reflexive reaction to the stimulation (Brown, 1911, 1914; Llinás, 2002). *Friedrich Goltz*, one of the critics of cerebral localization who believed that intellect cannot be localized within specific part of the cerebrum, also did not believe in the simplistic idea that the central nervous system and its behavior function on a purely reflexive basis (Finger, 2001). If we look at the history of EEG, *Hans Berger* was among the first scientists to describe spontaneous electrical activity in a relaxed resting state with the eyes closed (Berger, 1929). Another pioneer in electroencephalography, *Adolf Beck*, observed spontaneous electrical brain activity during his experiments with evoked potentials. Beck further described how spontaneous oscillations stop after stimulation and are not related to heart and breathing rhythms (Coenen and Zayachkivska, 2013). These two pioneers in electroencephalography were not the only ones to observe spontaneous electrical oscillations (see e.g., Coenen and Zayachkivska, 2013).

In the light of the above, it cannot be concluded that intrinsic activity brought a completely new idea about brain activity, which would radically break from the history of neuroscience and completely change our understanding of the brain. Therefore, it does not satisfy the second condition.

## Conclusion

Although intrinsic activity and the DMN have experienced great expansion and meet several of Kuhn's ideas, the idea that intrinsic activity is the new paradigm of neuroscience and a large scientific revolution, is untenable. However, the importance of intrinsic activity for the fMRI cannot be denied and therefore it may be claimed that the discovery of intrinsic activity was an *essential scientific revolution* for the field of fMRI. Intrinsic activity certainly changed our understanding of the brain and its activity; however, it cannot be considered as an all-changing scientific revolution that would replace reactive thinking about the brain, absolutely break from history of neuroscience, and determine a completely new view of brain functioning.

## Author contributions

MH wrote the review.

## Funding

This work was supported by Czech Science Foundation-Czechia (GACR) grant No. 17-23718S, and by the NPU I project “NPU4NUDZ” No. LO1611 from the MEYS CR.

### Conflict of interest statement

The author declares that the research was conducted in the absence of any commercial or financial relationships that could be construed as a potential conflict of interest.
